# A Brief Video-Based Intervention to Improve Digital Health Literacy for Individuals With Bipolar Disorder: Intervention Development and Results of a Single-Arm Quantitative Pilot Study

**DOI:** 10.2196/59806

**Published:** 2025-05-09

**Authors:** Emma Morton, Sahil S Kanani, Natalie Dee, Rosemary Xinhe Hu, Erin E Michalak

**Affiliations:** 1 School of Psychological Sciences Monash University Clayton Australia; 2 Department of Psychiatry University of British Columbia Vancouver, BC Canada; 3 Collaborative Research Team to Study Psychosocial Issues in Bipolar Disorder Vancouver, BC Canada

**Keywords:** mHealth, bipolar disorder, self-management, apps, digital health literacy, video-based intervention, bipolar, single-arm pilot trial, smartphone apps, mental health, psychological, quality of life, mood instability, effectiveness, acceptability, mental health apps, patient education, intervention

## Abstract

**Background:**

Smartphone apps can improve access to bipolar disorder (BD) care by delivering elements of effective psychological interventions, thereby promoting quality of life and reducing relapse risk and mood instability in BD. While many people with BD are interested in using publicly available mental health smartphone apps, without guidance, they risk selecting apps that are unsafe or ineffective.

**Objective:**

This study aimed to co-design a brief educational video on identifying appropriate mental health apps and to evaluate the acceptability and impact of this video among individuals with BD.

**Methods:**

Individuals with lived experience of BD, including 2 peer researchers and members of 2 advisory groups (n=4 and n=7), were consulted to develop a video with information on selecting safe, effective, and engaging mental health apps for BD. Video acceptability and impact on self-reported digital health literacy (including both general eHealth literacy and more specific mobile health literacy) were evaluated via a web-based survey, including both a validated measure and complementary items developed by the research team.

**Results:**

In total, 42 individuals with BD completed the evaluation survey (n=29, 69% women, mean age 38.6, SD 12.0 years). Digital health literacy, measured using the self-report eHealth Literacy Scale, significantly improved after viewing the video (pre: mean 32.40, SD 4.87 and post: mean 33.57, SD 4.67; t_41_=–3.236; *P*=.002; d=–0.50). Feedback supported the acceptability of the video content and format. Self-report items developed by the study team to assess mobile health literacy showed that individuals felt better able to determine which apps would protect their data (*P*=.004) and to ask their health care provider for support in choosing apps (*P*<.001) after watching the video.

**Conclusions:**

This study found preliminary evidence that an educational video can help people with BD improve their ability to identify, apply, and evaluate the quality of digital health resources. The video and a supplementary web-based educational module are freely available for implementation in health care settings and have the potential to be a cost-effective and accessible resource for clinicians to support patients with BD to navigate the public app marketplace in support of their self-management goals.

## Introduction

Bipolar disorder (BD) is a mental health disorder characterized by recurring periods of depressed or elevated moods, which can range in severity from mild mood elevation (BD type II; BD-II) to severely disruptive manic symptoms that may even necessitate hospitalization (BD type I; BD-I). Adjunctive psychological interventions for BD can delay episode recurrence and reduce symptom severity [1]. However, only 54% of individuals with BD receiving pharmacological treatment have accessed psychosocial services [2]. Smartphone apps could improve access to care by facilitating mood and sleep monitoring, providing psychoeducation, supporting medication adherence, and enabling in-the-moment application of coping skills [3] and may benefit quality of life, relapse risk, and mood instability in BD [4-6].

Unfortunately, research-led efforts to develop evidence-based mental health apps are rarely made publicly available. For example, a review of apps for psychosis found that only 15% of research apps were accessible on the public marketplace [7]. In contrast, there is a boom in commercial mental health apps [8,9]. The acceptability and uptake of apps in people with BD are high, with 77% expressing interest in receiving mental health treatment via their mobile device [10], and 42% reporting use of an app to support mood or sleep self-management [11].

There are drawbacks to consider in regard to the safety, efficacy, and feasibility of apps for BD. A review of the top 98 apps returned for the search term “bipolar” found that almost half were not clearly relevant to BD, no patient-facing apps were developed by a university or health care organization, and only 1 app had peer-reviewed literature to support its efficacy [12]. Two-thirds of apps offered privacy policies, of which 41% shared personal data with third parties. Some apps contained potentially harmful content such as advice misaligned with treatment guidelines and stigmatizing or triggering content. Further, the majority of apps for BD did not contain features to support user engagement, despite the fact that many commercial apps report poor user retention [13].

Given the variable quality of publicly available apps for BD, it is unsurprising that consumers experience challenges in selecting appropriate options. Results from an international survey regarding app use among people with BD found that younger age, education below a postgraduate level, and lack of experience using mood or sleep self-management apps were associated with lower levels of digital health literacy (the ability to identify, evaluate, and use health information in an online context) [14]. Individuals with lower health literacy are less likely to adopt eHealth resources or perceive them as useful while simultaneously overestimating the privacy protections offered by health apps [15]. As such, these groups are at risk of selecting unsafe or inappropriate apps (or conversely, not using potentially helpful apps).

Supporting informed decision-making in mental health app use through developing digital health literacy skills is necessary for an equitable digital mental health ecosystem [16]. Ideally, clinicians would play a role in referring individuals with BD to credible, safe, and engaging apps, given their role as a trusted information source [9,17]. In practice, a web-based survey of health care providers found that only 50% had discussed or recommended smartphone apps to patients with BD [18]. Alternative information sources accessible to patients include expert-reviewed app libraries, such as Psyberguide [19,20], the mHealth Index and Navigation Database [21,22], and the Organisation for the Review of Care and Health Apps [23]. Individuals with BD rarely sought information on health apps from such resources, preferring to seek recommendations from others with BD, app store reviews, or family or friends [14].

An alternative strategy to relying on health care provider recommendations or app libraries is to enhance digital health literacy skills in patients. One such intervention targeting people with serious mental illness is the 4-week Digital Opportunities for Outcomes in Recovery Services (DOORS) course [24]. However, the length and foundational content of this program (eg, basic smartphone functions) may not be suitable for all individuals with BD, given research showing people with BD have high levels of smartphone ownership [14] and higher digital health literacy than people with psychosis [25].

Brief videos may be an acceptable method to succinctly communicate key messages regarding mental health app selection and have previously been shown to be an effective knowledge translation strategy for people with BD [26]. They require a lower time commitment to learning than an in-person course such as DOORS and may be shared easily across a wide range of electronic devices (eg, phones and computers), potentially enhancing their reach and accessibility. Brief videos could also be embedded in psychological interventions for BD or provided as a supplementary resource, as a way to support individuals with BD to self-identify smartphone apps relevant to the self-management strategies taught in psychoeducation or in psychotherapy [3].

This study aimed (1) to develop a brief educational video describing strategies for selecting safe, effective, and engaging mental health apps and (2) to evaluate the acceptability and impacts of this intervention among people with BD.

## Methods

### Ethical Considerations

Ethics approval for the video evaluation was granted by the University of British Columbia Behavioral Research Ethics Board (H21-03767) on January 19, 2022. All participants received written information about the study and provided written consent before proceeding. Data in the study were treated confidentially and stored on a secure server in Canada. Participants were entered into a prize draw for 1 of 2 CAD $50 (approximately US $35) Visa gift cards. The authors assert that all procedures contributing to this work comply with the ethical standards of the relevant national and institutional committees on human experimentation and with the Helsinki Declaration of 1975, as revised in 2008.

### Study Design

#### Overview

The project was implemented across 2 phases. In the first phase, we applied principles of community-based participatory research (CBPR) to develop a brief video promoting awareness of the potential risks and benefits of mental health apps for individuals with BD and strategies to select appropriate apps. In the second phase, we conducted a quantitative evaluation of the acceptability and impact of the brief psychoeducation video.

#### CBPR Framework

The study was conducted using a CBPR framework: academic researchers or clinicians and those with lived experience worked in partnership to identify research priorities, conduct research, and disseminate findings [27]. The approach used was informed by 20 years of experiential knowledge of applying CBPR methods in BD research and knowledge translation by the Collaborative Research Team to Study Psychosocial Issues in Bipolar Disorder (CREST.BD) research network [28]. Details of the CREST.BD network are summarized below; a fulsome case study describing the network’s history and use of CBPR methods to determine network priorities has been previously published [29], along with papers describing the network’s approach to CBPR in a BD context [28,30].

The CREST.BD network was established in 2005 as a British Columbia–focused team of clinicians and researchers with expertise in BD and psychosocial treatments, with an emphasis on community-engaged research. In 2010, it expanded to a Canada-wide network and formally established advisory groups consisting primarily of individuals with lived experience of BD as well as clinicians and representatives of community organizations. Since then, the network has expanded its scope and geographic representation: team members specialize in a range of disciplines (ie, psychology, psychiatry, criminology, nursing, social work, gerontology, occupational therapy, and genetic counseling) and are located internationally, with particularly strong representation in the United States, the United Kingdom, and Australia. The current membership of CREST.BD can be viewed on the website [31]. Membership of the CREST.BD advisory groups has changed over the years, and project-specific advisory groups have also contributed to network activities. As some members are not publicly disclosed as living with BD, the identities of advisory group members are not detailed on the website.

**Figure 1:**
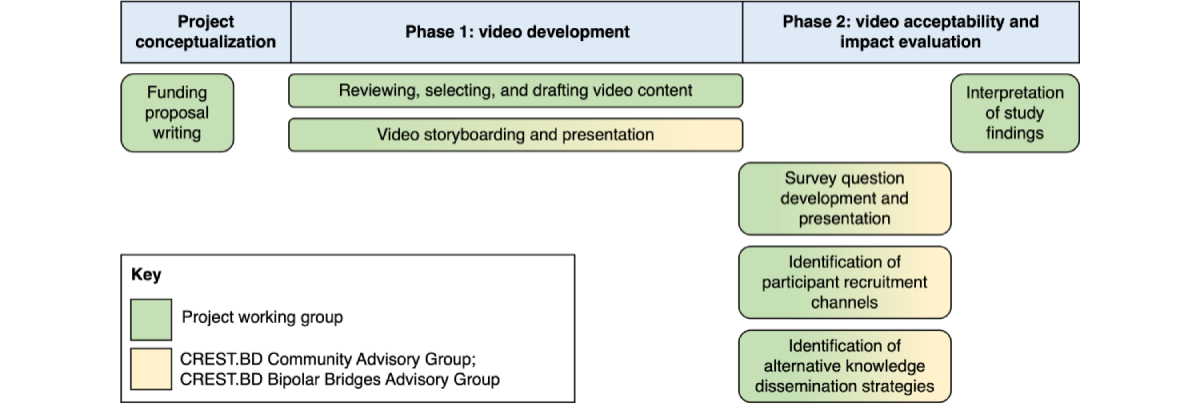
Involvement of lived experience and community perspectives across the project phases. CREST.BD: Collaborative Research Team to Study Psychosocial Issues in Bipolar Disorder.

In this work, CBPR activities were led by a subset of CREST.BD members (EM or EEM) and peer researchers through a project working group. In addition, 2 CREST.BD advisory groups were actively consulted on project activities. The membership of these groups and their involvement in the project, from conceptualization and funding acquisition through to the preparation of study findings, is summarized in Figure 1 and described further below.

#### The Project Working Group

Following the principles of CBPR, the video-based intervention was developed using the combined expertise of academic researchers, people with BD, and health care providers. The roles and experiences of all project working group members are described in detail in Table 1. The project working group met 4 times over Zoom (Zoom Video Communications) over the course of the project. Additional collaboration occurred asynchronously over email and shared Google Documents.

In this project, peer researchers were active members of the research team who drew on their lived experience of BD, and the unique sociocultural contexts they live and work in, to ensure the video and its corresponding evaluation aligned with the needs and values of people living with BD. Specifically, they contributed to the development of the funding proposal, selection and drafting of video content, consultation regarding video presentation, and interpretation of study findings. They also provided feedback on the evaluation study, including the selection and presentation of evaluation survey items and the identification of recruitment avenues. On the spectrum of public participation [32], the peer researchers were involved at the “collaborate” level; they contributed to all decisions regarding video content and presentation and informed the evaluation component. In recognition of their high degree of involvement, they are coauthors of this publication.

**Table 1:** Project working group membership.

Group member	Role	Relevant experiences
ND	Peer researcher	ND has 7 years of lived experience of BD^a^-II, and many more years of experience of being a supporter of someone living with BD. She has been a CREST.BD^b^ peer researcher since May 2020; she is a member of the PolarUs User Group and has contributed to writing content for the app. Along with her lived experience, she brought her experience in user experience and content design to the project.
RXH	Peer researcher	RXH is a Chinese immigrant who lives well with BD. She is a law student and was a member of CREST.BD advisory groups between 2020 and 2024.
EM	Academic or clinician	EM is a psychologist and researcher. At the time of this project, she was a postdoctoral fellow in the Department of Psychiatry at the University of British Columbia. Her research expertise lies in mood disorders, quality of life and patient-centered outcomes, psychosocial interventions, and digital mental health. She has been a CREST.BD member since 2015.
EEM	Academic	EEM is a professor in the Department of Psychiatry at the University of British Columbia. Her research expertise lies in mood disorders, digital mental health, patient engagement in research, knowledge translation, quality of life, and global mental health. She is the founder and network lead of CREST.BD.

^a^BD: bipolar disorder.

^b^CREST.BD: Collaborative Research Team to Study Psychosocial Issues in Bipolar Disorder.

#### Consultation With CREST.BD Advisory Groups

Two CREST.BD advisory groups were actively consulted on the content and delivery of the video, the selection and presentation of evaluation survey items, and the identification of recruitment avenues. One advisory group (Community Advisory Group) consulted at a high level on the network’s program of research and was primarily comprised of people living with BD; other group members were a clinician, representatives of community organizations, and a community engagement and knowledge translation coordinator with a specialty focus on diverse and marginalized communities [29]. The other advisory group (Bipolar Bridges Advisory Group) consulted specifically on the development of an app for BD and was comprised only of people with lived experience of BD [33]; feedback was therefore obtained from individuals with varying degrees of interest in and familiarity with apps. Membership of the Bipolar Bridges Advisory Group specifically privileged individuals of diverse genders, sexual orientations, ethnicities, and cultural backgrounds.

Here, the advisory groups provided feedback on specific decisions about the video content and presentation and the evaluation strategy (including questionnaire wording and recruitment avenues). The groups also generated new ideas for alternative knowledge dissemination strategies that were the focus of later development efforts (see Discussion section). The advisory groups were consulted on 3 occasions over Zoom over the course of the project (attendance ranged from n=4 to n=7). Additional feedback was obtained asynchronously via email. On the spectrum of public participation [32], the advisory groups contributed at both the “consult” and the “involve” level in the context of their longstanding contributions to establishing the CREST.BD strategic plan, research priorities, and ways of working, a process that has been documented in detail elsewhere [28]. All members of the advisory groups share the same scope of decision-making power.

### Phase 1: Development of the Video

#### Overview

Video development occurred between October 2021 and December 2022. Key messages and strategies for the video content were informed by the working group collaboratively reviewing and discussing existing resources (eg, the mHealth Index and Navigation Database and the DOORS curriculum [22,24]), research on specific digital health needs of people with BD and depression [34,35], and peer researcher reflections on their own lived experiences. The script was then drafted by EM and revised with input from EEM, ND, and RXH. Peer researchers were also involved in facilitating consultations with the CREST.BD advisory groups regarding the draft script and storyboard, with feedback integrated into the final video. Decisions regarding video look and feel were driven by peer researchers ND and RXH, who reviewed mood boards and previous videos by the artist to inform decisions regarding video presentation.

**Figure 2:**
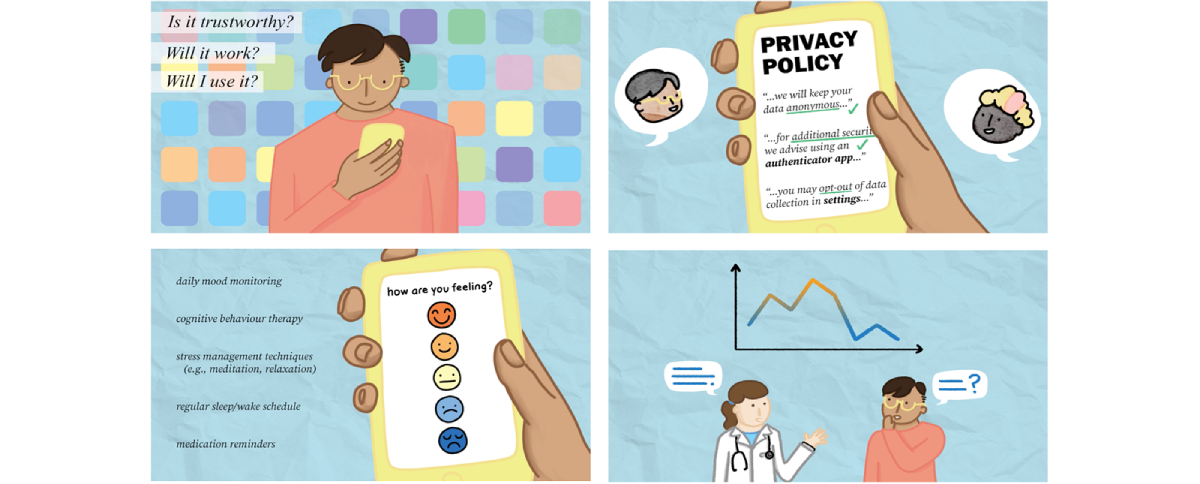
Stills from the video-based intervention illustrating topics covered including assessing privacy and security, use of evidence-based techniques, and ease of use.

The guiding principles for video presentation were collaboratively decided by the project working group: the aim was to keep the video short, simple, and informative to make it easy for people living with BD to understand and apply the recommendations. Reflecting the values expressed by peer researchers, we deliberately targeted a wide range of patient demographics, and accessibility concerns (eg, cognitive difficulties, color blindness, hearing problems, and English as a second or foreign language) were considered in script development, storyboarding, and dissemination plans. For example, we used representative images rather than text wherever possible to minimize demands on working memory and facilitate subtitling and translation (Figure 2). The final video can be viewed on YouTube [36], and the script is available in Multimedia Appendix 1.

#### Video Content

##### Overview

The video content was informed by key app evaluation frameworks, in combination with previous research (both specific to BD and relevant to the use of apps in other populations), and refined through repeated consultation with peer researchers and the CREST.BD advisory groups. Broad topic areas addressed in the video were informed by the American Psychiatric Association (APA) app evaluation model, which in itself was developed by harmonizing 45 different app evaluation frameworks [37,38], and consist of five different levels: (1) background information (eg, cost, accessibility, developer information, and system requirements), (2) privacy and security (eg, availability of a privacy policy, collection and use of data, data protection, and management of safety risks), (3) evidence base (eg, clinical foundation and evidence of efficacy or feasibility), (4) ease of use (eg, usability and engagement features), and (5) data integration. Video content centered on privacy and security, evidence base, and ease of use, as there is growing consensus between approaches to app evaluation that data security measures and clinical foundations are of central importance [39,40]. Similarly, engagement with content and features is necessary for apps to have beneficial effects [41,42]. The decision to emphasize these topics is reinforced by data, showing that people with BD report content quality or accuracy, ease of use, and control over information privacy or security among the top 4 most important mental health app features [34]. Specific recommendations relevant to each chosen level of the APA app evaluation model are informed by the following considerations:

##### Privacy and Security

We represented mHealth Index and Navigation Database criteria deemed essential by a previous review [22,43]: having a privacy policy, reporting security measures, declaring data use and purpose, allowing for the deletion of data, and allowing users to opt out of data collection. Feedback from peer researchers was that difficulties in interpreting the complex regulatory language of privacy policies should be normalized and that viewers could be directed to look for key phrases or to seek additional help from health care providers.

##### Evidence Base

To support viewers in evaluating the clinical foundations of an app, we described features with the potential to facilitate key mediating mechanisms of evidence-supported psychosocial interventions [3]. In addition, feedback from peer researchers was that peer-reviewed literature is often difficult for a layperson to access or understand and that viewers should be encouraged to seek support from health care providers in reviewing research evidence.

##### Ease of Use

We highlighted features with the potential to support engagement (notifications, meaningful use of self-monitoring data, and gamification elements like streak counters), drawn from an international survey of people with BD [34]. Based on prior research on barriers to app engagement in people with a mood disorder [34,35], as well as feedback from peer researchers, we strove to normalize BD-related fluctuations in mood and energy and their consequent impacts on engagement.

### Phase 2: Evaluation of the Video-Based Intervention

#### Overview

Evaluation of the video-based intervention was conducted using the web-based Qualtrics platform. Participants provided demographic information, completed baseline assessments, viewed the video, and responded to evaluation items immediately afterward. Data collection occurred between February and October 2023.

#### Participants and Recruitment

Participant recruitment occurred via promotion on CREST.BD social media pages, paid advertisements on Facebook, Instagram, and Twitter, emails to the CREST.BD mailing list, and health care providers or organizations associated with the CREST.BD network (eg, Hope+Me, a Toronto-based community organization offering peer support and counseling; Bipolar Support Club International, an online, peer-led organization offering support and education; and the John Hopkins Bipolar Disorder clinic, an academic psychiatry center offering BD-specific consultation and care). CREST.BD network members based internationally (including academics, clinicians, representatives of mental health advocacy organizations, people with lived experience of BD, and caregivers or supports of individuals with BD) were invited to disseminate the recruitment materials through their networks.

Inclusion criteria were (1) age 19 years or older, (2) a self-reported diagnosis of BD, and (3) access to a personal smartphone device. The evaluation survey was open internationally.

### Data Collection

#### Overview

A web-based survey was developed based on previous literature and refined through peer researcher and advisory group input (Multimedia Appendix 2). At baseline, individuals were asked to provide information on demographics (age, gender, cultural and racial background, education, and occupation), clinical characteristics (BD diagnosis and current treatment), and technology use (use of self-management apps and preferred information sources). Questions related to eHealth literacy and mobile health (mHealth) literacy (described below) were asked before and after viewing the video. After the video, 6 Likert-scale statements developed by the researchers (EM or EEM) were used to obtain video acceptability ratings (1=strongly disagree to 5=strongly agree).

#### eHealth Literacy

The eHealth Literacy Scale (eHEALS) was used to evaluate self-assessed knowledge and confidence in identifying, applying, and evaluating the quality of digital health resources [44]. Eight self-report Likert-type items (1=strongly disagree to 5=strongly agree) are summed to create an overall score (range 8-40), with higher scores indicating greater digital health literacy. Two additional Likert-type items assess respondents’ perception of the utility and importance of digital health resources; these are not included in the overall score calculation. The 1-factor structure and reliability of the eHEALS have been demonstrated in the general population [44-46] and populations with health conditions [47-49].

#### mHealth Literacy

While the eHEALS is the most commonly used measure of digital health literacy [50], it was developed prior to the widespread use of apps and therefore may not encompass all relevant aspects of mHealth literacy. To address this, 6 additional items (using the same 5-point Likert scale as the eHEALS) were developed by the researchers (EM or EEM) to assess self-perceived knowledge and confidence specific to searching for, evaluating, and using self-management apps (Multimedia Appendix 2). These items were not validated.

### Data Analysis

Data were analyzed using SPSS (version 29; IBM Corp). Descriptive statistics were used to summarize demographics and feedback regarding video acceptability. Paired-sample *t* tests were used to compare summary scores on the eHEALS before and after viewing the video. The ordinal nature of mHealth literacy items warranted the use of a nonparametric, 2-sample paired sign test to assess video impacts. Significance was set at *P=.*05, and all analyses were 2-tailed. Effect sizes for paired-sample *t* tests were estimated using Cohen *d*, and effect sizes for nonparametric, 2-sample paired sign tests were estimated using Cliff δ, given the nonnormal distribution of the difference scores [51,52]. Sensitivity analyses (Multimedia Appendix 3) were conducted to evaluate the potential influence of key demographic and baseline variables on missing data, the impact of outliers, and the influence of missing data [53].

## Results

### Survey Sample

Of individuals who consented to the survey (n=77), suspected fraudulent responses (n=23) were removed based on indicators including duplicate IP addresses, email addresses that did not match provided names, infeasible completion times, and duplicate responses to open-ended survey items [54,55], leaving 54 valid entries. In total, 42 respondents completed the survey; their data were used for analyses of acceptability and changes in digital health literacy.

Demographics are summarized in Table 2. Survey completers were primarily women (n=29, 69%), White (n=31, 74%), and residing in North America (n=34, 81%), with a mean age of 38.6 (SD 12) years. Under half the sample self-reported a BD-II diagnosis (n=19, 45%), and most participants were receiving psychiatric treatment, including medication (n=38, 90%) and counseling (n=25, 60%). The majority of the sample had completed postsecondary education (n=34, 81%).

To provide some insights into whether data were missing in a systematic fashion (Multimedia Appendix 3), we compared those who dropped out prior to survey completion and those who completed the study using independent *t* tests for age and baseline eHEALS. Chi-square tests were used to assess for differences in survey completion rates related to gender and previous use of BD-related health apps, as this was found to be associated with digital health literacy in a previous analysis [14]. We did not assess for differences between BD-I and BD-II, as in the same previous analysis, when BD-I was used as the reference category in our regression model BD-II did not emerge as a significant predictor of eHEALS scores [14]. No significant differences were found between completers and noncompleters, suggesting that missing data were not associated with these demographic characteristics.

**Table 2:** Demographic and clinical characteristics of survey participants.

Demographic or clinical variable		Total sample (N=54)	Survey completers (n=42)
Age (years), mean (SD)		40.1 (12.0)	38.6 (11.8)
**Gender,** **n** **(%)**			
	Woman	35 (65)	29 (69)
	Man	15 (28)	10 (24)
	Nonbinary or gender nonconforming	3 (6)	2 (5)
	Other or prefer not to answer	1 (2)	1 (2)
**Country or region of residence,** **n** **(%)**			
	Canada	24 (44)	20 (48)
	United States	19 (35)	14 (33)
	United Kingdom and Northern Ireland	5 (9)	4 (10)
	Asia	3 (6)	2 (5)
	Africa	2 (4)	1 (2)
	Australia	1 (2)	1 (2)
**Race or ethnicity,** **n** **(%)**			
	Asian	4 (7)	3 (7)
	Black	5 (9)	3 (7)
	Hispanic	2 (4)	2 (5)
	White	39 (72)	31 (74)
	Multiple ethnicities	3 (6)	2 (5)
	Other or prefer not to answer	1 (2)	1 (2)
**Highest level of education,** **n** **(%)**			
	Did not finish high school	1 (2)	0 (0)
	High school	1 (2)	1 (2)
	Did not finish postsecondary	9 (17)	7 (17)
	Postsecondary diploma or certificate or associate degree	7 (13)	5 (12)
	Undergraduate (bachelor degree)	25 (46)	19 (45)
	Master degree or doctorate (PhD)	11 (20)	10 (24)
**Employment status,** **n** **(%)**			
	Employed full-time	21 (39)	16 (38)
	Employed part-time or casual	17 (31)	15 (36)
	Student	5 (9)	4 (10)
	Not in paid employment	7 (13)	4 (10)
	Retired	4 (7)	3 (7)
**Marital status,** **n** **(%)**			
	Single	21 (39)	18 (43)
	Committed or common-law relationship	13 (24)	10 (24)
	Married	12 (22)	10 (24)
	Divorced or separated	5 (9)	2 (5)
	Other or prefer not to answer	3 (6)	2 (5)
**BD** ^a^ **diagnosis,** **n** **(%)**			
	BD-I	26 (48)	21 (50)
	BD-II	24 (44)	19 (45)
	Other or do not know	4 (7)	2 (5)
Receiving treatment for BD, n (%)		50 (93)	40 (95)
**Type of treatment,** **n** **(%)**			
	Pharmacological	48 (89)	38 (90)
	Counseling or psychotherapy	28 (52)	25 (60)
	Peer support	7 (13)	6 (14)
	Other	2 (4)	1 (2)
**Previous use of apps for BD,** **n** **(%)**			
	Yes	29 (54)	24 (57)
	No	25 (46)	18 (43)

^a^BD: bipolar disorder.

### Video Acceptability

**Figure 3:**
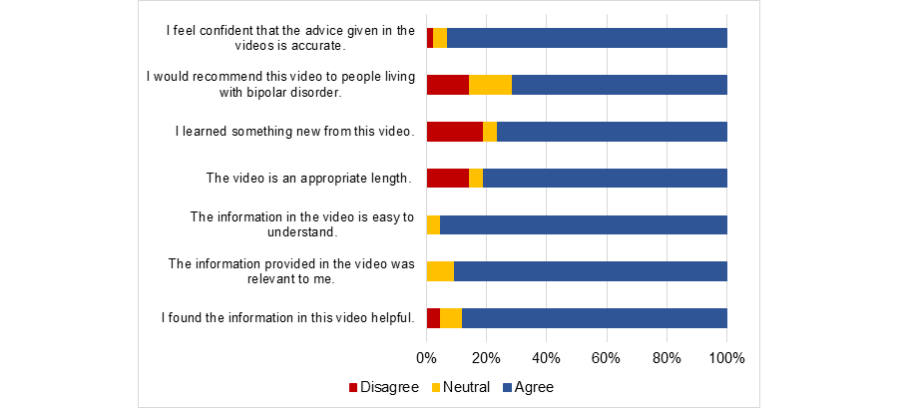
Survey completers’ responses (disagree or neutral or agree) to 6 survey questions evaluating video acceptability.

Perceptions of the content, length, and presentation of the video were overall positive (Figure 3). Ratings of video acceptability were collapsed to simplify the presentation (strongly agree or agree=agree and strongly disagree or disagree=disagree).

### Changes in eHealth Literacy

A paired-sample *t* test was used to assess the impacts of the video on eHEALS scores. No evidence of nonnormality was detected according to the Shapiro-Wilk test (*W*=0.96; *P=.*11) nor visual examination of the histogram and quantile-quantile plot. eHEALS scores of the survey completers were significantly higher after watching the video (mean 33.57, SD 4.67) than at baseline (mean 32.40, SD 4.87; t_41_=–3.236; *P=*.002; *d*=–0.50). The influence of 2 potential outliers was evaluated via a paired-sample *t* test with outliers removed. As overall findings remained unchanged (Multimedia Appendix 3), these cases were retained.

For a conservative estimate of the impact of missing data [53,56], the paired-sample *t* test was repeated with posttest data for survey noncompleters imputed using the last observation carried forward. Results from this sensitivity analysis showed a significant improvement in eHEALS scores after viewing the video (Multimedia Appendix 3).

### Changes in mHealth Literacy

Responses of survey completers to mHealth literacy items before and after viewing the video are summarized in Table 3. A Shapiro-Wilk test showed that the distribution of the difference scores of evaluation items departed significantly from normality (question 1: W=0.74; *P*<.001; question 2: W=0.70; *P*<.001; question 3: W=0.87; *P*<.001; question 4: W=0.88; *P*<.001; question 5: W=0.92; *P*=.007; and question 6: W=0.77; *P*<.001). Distributions of the difference scores were found to be nonsymmetrical from visual inspection of the histograms.

Based on the skewed and nonnormal distribution of the differences, a nonparametric, 2-sample paired sign test was used to evaluate changes in participant responses to mHealth literacy items (Table 3). Positive differences indicate the number of cases where responses were higher after watching the video compared to before. Negative differences indicate the number of cases where responses were lower after watching the video than before. Ties indicate no change in ranking. After watching the video, survey respondents felt better able to determine which apps would protect their data (*P*=.004; δ=.417) and were more empowered to ask their health care provider for support in choosing an app (*P<*.001; δ=.253). The median response to these items changed from neither agree nor disagree to agree.

**Table 3:** Median rankings and 2-sample paired sign test results comparing respondent’s ranking of mobile health (mHealth) literacy items before and after watching the video-based intervention^a^.

mHealth literacy item	Survey completers (n=42)						
	Median prevideo (IQR)	Median postvideo (IQR)	Positive differences, n (%)	Negative differences, n (%)	Ties, n (%)	*P* value (2-tailed)	δ
Question 1: I know how to use smartphone apps to optimize my health and well-being.	5.00 (4.00-5.00)	4.00 (4.00-5.00)	5 (12)	13 (31)	24 (57)	.096	–0.130
Question 2: I feel motivated to use smartphone apps to optimize my health and well-being.	4.00 (4.00-5.00)	4.00 (4.00-5.00)	5 (12)	8 (19)	29 (69)	.58	–0.0306
Question 3: I am able to find and download a mental health app that fits my needs.	4.00 (3.00-5.00)	4.00 (3.00-5.00)	13 (31)	8 (19)	21 (50)	.38	0.0459
Question 4: I am able to differentiate between apps that protect my data and apps that do not.	3.00 (2.00-4.00)	4.00 (3.00-4.00)	20 (48)	5 (12)	17 (40)	.004	0.417
Question 5: I am aware of resources that can help me evaluate mental health apps.	4.00 (3.00-4.00)	4.00 (3.00-5.00)	17 (40)	7 (17)	18 (43)	.06	0.223
Question 6: I am able to ask my health care provider for support with finding and evaluating mental health apps.	3.00 (2.00-4.00)	4.00 (2.00-4.00)	17 (40)	2 (5)	23 (55)	<.001	0.253

^a^Items are scored on a 5-point Likert scale, where 1=strongly disagree, 2=disagree, 3=neither agree nor disagree, 4=agree, and 5=strongly agree.

## Discussion

### Principal Findings

With the input of people living with BD, we developed a brief psychoeducational video designed to support individuals with this condition in selecting safe, effective, and engaging mental health apps. Preliminary evaluation data show that the video was largely perceived as acceptable, and viewing the video resulted in improvements to eHealth literacy. This study adds to a body of research showing that educational initiatives can improve digital health literacy for people with chronic health conditions. A previous scoping review identified 9 interventions aimed at improving digital health literacy that were grouped into 2 categories: those providing education and training and those providing social support, with education and training initiatives (including videos, workshops, and massive open online courses) showing greater benefits for digital health literacy [50]. We are only aware of 2 interventions developed to address digital health literacy in individuals with mental health conditions, including DOORS (developed to support individuals with psychosis to use smartphones and apps) [24] and video-based training to use a patient portal for people with chronic conditions (including depression and anxiety, among other physical health conditions) [57]. While these interventions reported positive effects for eHealth literacy measures, neither were developed with specific consideration of the app-related preferences and information needs of people living with BD, a gap addressed by our video-based intervention.

To complement the eHEALS, which is focused on digital health literacy more broadly, we also included some researcher-developed items to evaluate change in smartphone-specific competencies, such as searching for and evaluating apps. Positively, we observed improvements to some aspects of mHealth literacy, such as willingness to ask a health care provider for support and confidence in evaluating app privacy policies. We note that our previous web-based survey of health care providers found a common barrier to discussing or recommending smartphone apps to patients with BD was practitioner knowledge [18]—our findings therefore suggest that clinician education efforts are also needed in order for patients to receive the desired support from health care providers regarding app selection. Furthermore, in light of consensus that the presence of privacy and data security protections is of foundational importance in the decision of whether or not to use apps [39,40], and BD-specific literature showing control over information privacy or security ranks among the top 4 most important mental health app features [34], the finding that confidence evaluating privacy policies improved after the video is of particular note. As we included several strategies to support viewers in evaluating privacy policies (ie, key aspects of privacy policies, encouragement to seek the support of health care providers, and links to app libraries), future qualitative evaluations could explore which of these were most impactful from a viewer perspective, which could inform refinements to this and similar digital health literacy interventions.

It is important to acknowledge that not all aspects of mHealth literacy demonstrated improvements. Potentially, this may be indicative of some ceiling effects, given median baseline responses to items that did not demonstrate change were “agree” or “strongly agree.” We acknowledge the possibility that the use of web-based recruitment methods may have biased the participating sample to individuals with higher baseline digital health literacy as well as interest in app-based tools (described further in the Limitations section). However, it is also possible that the brief video-based intervention was not detailed enough to result in changes to self-perceived knowledge. Indeed, while video acceptability ratings were overall positive, some minor disagreement was observed regarding the appropriateness of the length of the video. Our own CREST.BD advisory groups offered similar reflections regarding the need to offer more in-depth learning opportunities for specific subgroups; the development of a suite of self-guided educational resources to address this feedback is detailed below.

**Figure 4:**
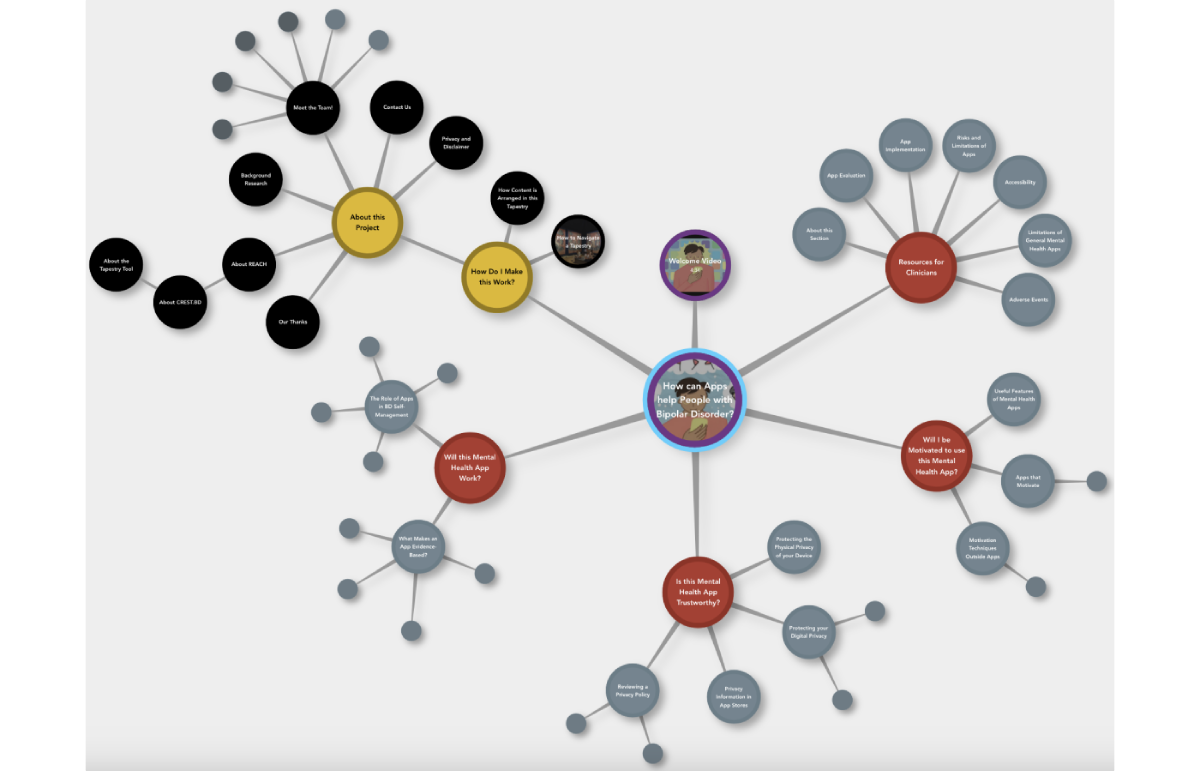
Navigation structure of the Tapestry Tool educational module containing resources for people with BD and health care providers (to view module content, please visit [62]). BD: bipolar disorder; CREST.BD: Collaborative Research Team to Study Psychosocial Issues in Bipolar Disorder.

Our project adds to a body of literature on the utility of CBPR frameworks for developing educational outputs that are well-received and impactful in the target population [58-60]. Input from peer researchers and advisory groups helped to ensure that the video focused on issues of primary importance to people with BD, that recommendations were feasible and practical, and that video delivery was engaging and accessible. Participatory research activities in this study also highlighted challenges in planning the timelines and scope of projects developing and evaluating interventions using CBPR frameworks. For example, discussion with peer researchers and advisory groups identified potential user groups whose needs may not be sufficiently met by the intervention as originally conceptualized (ie, a brief video). It was noted that specific subgroups, such as those impacted by the digital divide, may need guidance in basic phone features or additional resources to support the application of strategies. The informational needs of health care providers were also highlighted via consultation activities and a prior survey [18]. To address this feedback, coauthors EM, EEM, and SSK created a complementary suite of self-guided resources for people with BD and health care providers, structured around the video themes (ie, privacy, efficacy, and engagement) and levels of the APA app evaluation framework not covered in the video (ie, background information and data integration). Emerging information regarding the potential risks of apps in BD, such as the potential for mood monitoring to reinforce depressive symptoms in vulnerable individuals [61] and the limitations of using apps designed for the general population for BD concerns [11], was also detailed. These resources were hosted on an innovative learning platform, the Tapestry Tool [62], where hierarchical relationships between concepts are represented spatially similar to a mind map (Figure 4), and multimodal resources including text, videos, and web articles can be linked. Similar online courses to support digital health literacy have been shown to improve eHEALS scores in specific populations, such as people with type 1 and 2 diabetes [63]. Combining this brief video with a self-guided exploration of the Tapestry Tool educational module could therefore further enhance impacts on digital health literacy. However, as this Tapestry Tool educational module was developed in addition to the planned, funded activities (ie, development of the brief video), we did not have the resources to evaluate the impacts of these resources separately and in combination. This illustrates a common tension in CBPR research: extensive consultation with communities is needed to inform grant applications; yet, this can be difficult to resource before grant funding is available [64]. To avoid situations where there are not sufficient resources to fund research priorities identified by the community, we suggest a need for more funding opportunities specifically supporting CBPR during project conceptualization.

### Limitations

A number of limitations to this study should be noted. For context, we note that the grant provided to fund this project (Michael Smith Health Research BC REACH Grant) was specifically intended to cover costs associated with the development of the educational resources (including payment of peer researchers). For this grant, costs associated with research studies are noneligible expenses and were covered in kind by CREST.BD. This limited our ability to conduct a more fulsome randomized controlled trial, as we did not have sufficient funds to fairly compensate participants for their involvement in a study where they may not have received exposure to the intervention. In addition, it limited our ability to conduct more resource-intensive recruitment strategies, such as outreach into face-to-face settings. The implications of this for the study limitations are described in more detail below.

First and foremost, this was a nonrandomized pilot evaluation; findings should therefore be interpreted with caution. In the absence of a control group, spontaneous improvements due to expectancy effects, baseline sample characteristics, or other confounding variables cannot be ruled out. In addition, the small sample size limits generalizability. Removal of suspected fraudulent responses detected on review of the data (n=23) reduced the total valid survey entries (n=54). This finding emphasizes the importance of applying additional strategies to ensure sample validity, such as rigorous screening procedures, inclusion of questions to detect poor quality or inattentive responses, and restrictions on where and how surveys are advertised [65]. Although our sample was small, it is comparable to other evaluations of digital health literacy interventions in serious mental illness populations [24,66]. Unfortunately, this sample was too small to conduct additional subgroup analyses, including gender-based comparisons.

Our sample was predominantly White and had completed some form of postsecondary education; efforts are needed to ensure that digital health literacy interventions are accessible to those with limited English proficiency. A survey of established (living in Canada for >10 years) senior Punjabi and Chinese immigrants (n=896) found that only one-quarter of participants reported advanced reading and writing proficiencies in English, and lower levels of education were associated with poorer eHEALS scores. As 65% of participants expressed an interest in using a smartphone to improve their health [67], this group may benefit from support to develop digital health literacy. To support equitable access to intervention content in Canada, we have translated the video into Mandarin, Punjabi, and American Sign Language, although we note that the evaluation was only conducted in English, limiting ability to generalize findings to other language groups.

Funding restrictions and issues of feasibility influenced our choice of recruitment strategy: we used a web-based survey to increase the likelihood of reaching a target sample size, given the relatively low prevalence of BD [68]. It may be that the use of web-based recruitment methods biased our sample toward individuals with higher pre-existing levels of digital health literacy. Relatedly, one survey that used telephone, hard-copy, and online data collection methods to assess digital health literacy and digital engagement for people with severe mental illnesses (including BD) found that higher levels of digital health literacy were associated with having outstanding or good self-reported knowledge of the internet [25]. As such, future studies should consider evaluating the impact of this video-based resource using alternative dissemination methods, such as DVDs that can be played in mental health clinics, or one-on-one consultations with health care providers.

The eHEALS measures self-perceived digital health literacy and not necessarily the actual performance of these skills; it is therefore possible that participants may experience an increase in self-perceived competencies without a concordant improvement in the real-world application of their skills. Future studies may wish to use procedural assessments of digital health literacy competencies. Approaches to performance-based assessments of digital health literacy are highly heterogenous and include simulated behavioral tasks, knowledge assessments, and evaluation tasks [69]. For example, previous studies have provided participants with a list of both high- and low-quality health information websites [70,71]; the concordance of participants’ evaluation of these websites with researcher ratings (as based on a standardized framework) was used to evaluate eHealth literacy skills. A similar approach could be used in the future to compare participants’ evaluations of apps with expert ratings as a proxy for mHealth literacy skills. Alternatively, comparing eHEALS scores to skills-based assessments may improve confidence about the real-world implications of improvements on this measure. While some work has been conducted to demonstrate modest correlations between perceived and performed eHealth literacy [72], we acknowledge that additional external validation is required. Unfortunately, we are not aware of any validated measures of mHealth literacy (performance-based or self-assessment)—a clear priority for future research. Our own in-house items were developed, given the dearth of available instruments; however, the fact that they were not validated remains a limitation of this study.

### Conclusions

Interventions are needed to help address the digital divide by promoting the skills and knowledge needed to take advantage of digital mental health tools and enhance the uptake of safe and effective mental health apps by people with BD. In this study, receiving only 4.5 minutes of psychoeducation about the risks and benefits of mental health apps for BD was found to improve self-perceived eHealth literacy and some aspects of mHealth literacy in individuals with this diagnosis. However, it must be noted that multiple aspects of mHealth literacy remained unchanged, and 19% (n=8) of the survey completers denied learning anything new as a result of the video. While findings remain preliminary due to the small sample size, nonrandomized design, and the use of nonvalidated mHealth literacy items, they are encouraging for future evaluations. To support the reach of the video and the accompanying web-based educational module, we have made these resources freely available for health care providers and patients [36,62].

## Appendix

Multimedia Appendix 1 “Choosing a bipolar disorder app that works for you” script.

Multimedia Appendix 2 Video-based intervention evaluation surveys.

Multimedia Appendix 3 Sensitivity analyses.

## Abbreviations

APA: American Psychiatric Association
BD: bipolar disorder
CBPR: community-based participatory research
CREST.BD: Collaborative Research Team to Study Psychosocial Issues in Bipolar Disorder
DOORS: Digital Opportunities for Outcomes in Recovery Services
eHEALS: eHealth Literacy Scale
mHealth: mobile health

## Data Availability

The datasets generated or analyzed during this study are not publicly available in accordance with ethics approval given by the ethics board from the participating university but are available from the corresponding author on reasonable request.
