# Supplementary material for: A Brief Video-Based Intervention to Improve Digital Health Literacy for Individuals With Bipolar Disorder: Intervention Development and Results of a Single-Arm Quantitative Pilot Study
Source: J Particip Med. 2025 May 9;17:e59806. doi: 10.2196/59806 (PMC12102627; doi:10.2196/59806)
Supplement: Multimedia Appendix 1 [file jopm_v17i1e59806_app1.pdf]

The full video may be viewed online on Youtube.

1	2	3
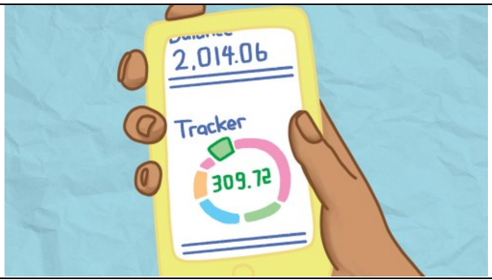	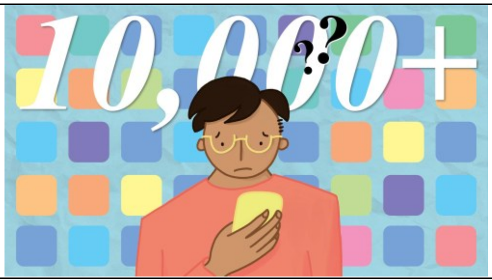	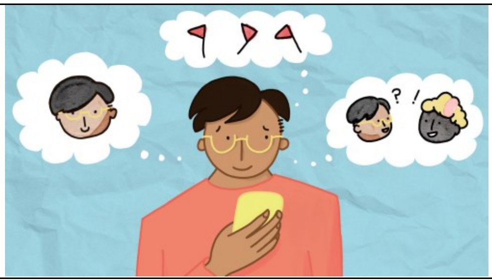
**Action:** A close up of a hand holding a smartphone and scrolling through a budgeting, fitness, and social media app.<br><br>**Voiceover:** Smartphone apps can help us with many aspects of our daily lives, from banking, to exercise, to staying in touch with family and friends.	**Action:** A close up of a person smiling and holding their mobile phone. Their smile turns into a concerned expression as tiles (representing apps) populate the background, and the numbers '10,000' appear behind them.<br><br>**Voiceover:** For those of us who live with bipolar disorder, apps can also be a tool to help empower us as we manage our mental health. Deciding which app to use, though, can be challenging **-** there are currently over 10,000 mental health apps, and new options are being added every day.	**Action:** A close up of a person holding their mobile phone. Their expression becomes more relaxed. As the questions are narrated, thought bubbles appear behind them with images to represent themselves, red flags, and a conversation with another person.<br><br>**Voiceover:** Choosing the right app for you involves a process of reflecting on your own needs and interests, keeping a lookout for any red flags, and maybe talking to your healthcare provider or a trusted supporter. There are three important questions to consider…

4	5	6
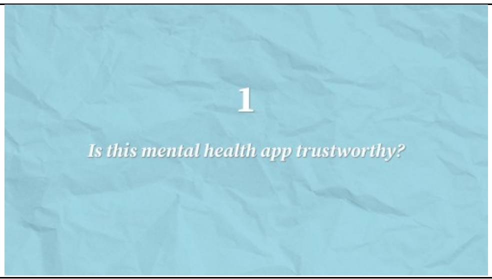	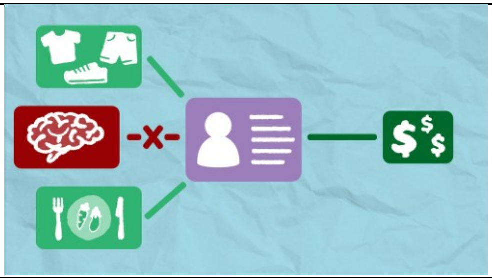	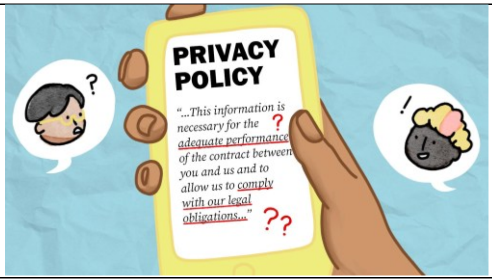
**Action:** A title card appears on a blank screen.<br><br>**Voiceover:** 1. Is this mental health app trustworthy?	**Action:** Icons representing types of personal data are depicted in a flow chart with dollar signs at the end. Clothing and food preferences are depicted in green and mental health data is depicted in red.<br><br>**Voiceover:** In the internet age, many of us have gotten used to the idea that our personal data is used to sell us various products and services.<br><br>However, while we might be comfortable with companies knowing our clothing and food preferences, our mental health data is a different story.	**Action:** A close up of a hand holding a smartphone and scrolling through an app privacy policy, with relevant text underlined. Two floating heads look on.<br><br>**Voiceover:** An app's privacy policy can tell you if they will keep your data private and secure.<br><br>The language used in privacy policies is often complicated and can be difficult to understand. If you're confused by an app's privacy policy, consider asking a trusted person to review it with you.<br><br>An app is more likely to be trustworthy if they promise to keep your data anonymous, offer data security measures, and allow you to delete your data or opt out of data collection entirely.<br><br>Be careful if a developer says they'll sell your health data to a third party. Be especially cautious if an app has no privacy policy at all!

7	8	9
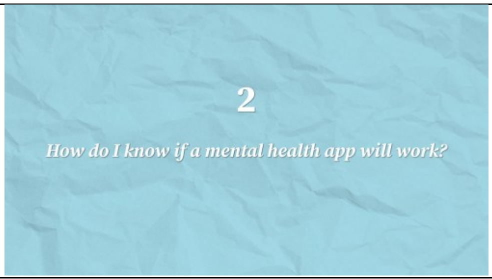	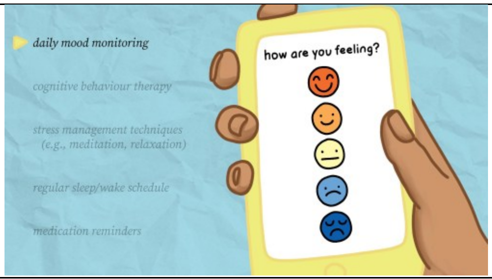	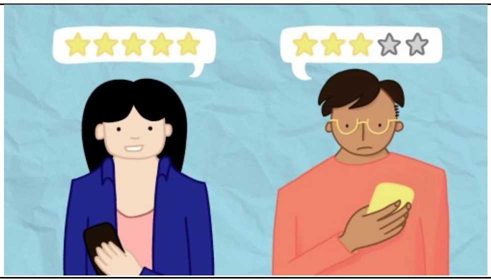
**Action:** A title card appears on a blank screen.<br><br>**Voiceover:** Another important question is, "#2 How do I know if a mental health app will work?"	**Action:** A close up of a hand holding a smartphone and scrolling through various types of mental health app<br><br>**Voiceover:** Apps tend to be more credible if they were developed by university or healthcare agencies.<br><br>You can also look at whether the app includes techniques that are evidence-supported for bipolar disorder. Things to look for include: daily mood monitoring; cognitive behaviour therapy; stress management techniques, like meditation or relaxation; keeping a regular sleep/wake schedule; or medication reminders.	**Action:** A close up of two people holding their mobile phones. Above their head are speech bubbles containing star ratings.<br><br>**Voiceover:** Positive user reviews can be helpful, but keep in mind that one person's experience of an app might not be the same as yours.

10	11	12
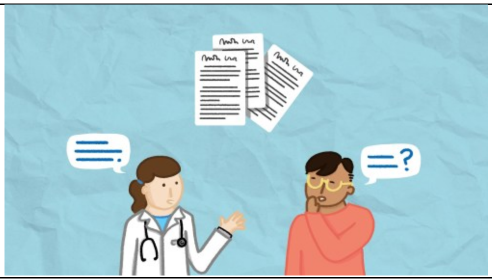	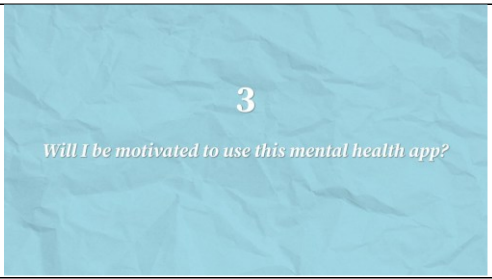	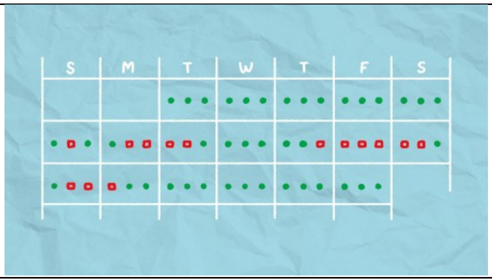
**Action:** A close up of a person talking to their doctor (indicated by speech bubbles) as a stack of papers appears overhead.<br><br>**Voiceover:** The most solid form of evidence is scientifically reviewed research that shows an app reduces bipolar disorder symptoms. Sometimes this research is not publicly available or is difficult to interpret - you can ask your healthcare provider to help you investigate.	**Action:** A title card appears on a blank screen.<br><br>**Voiceover:** 3. Will I be motivated to use this mental health app?	**Action:** A weekly calendar appears and is populated by green and red circles.<br><br>**Voiceover:** Research shows that using a mental health app consistently helps you get the most out of it. That said, it is still normal for your interest to ebb and flow depending on what's going on in your life, or your mental health symptoms - especially low mood.

13	14	15
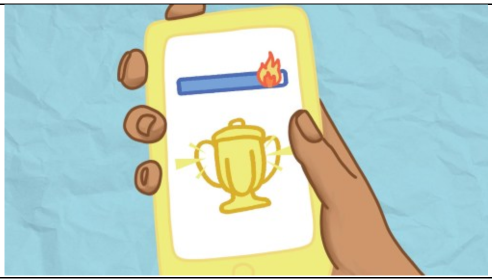	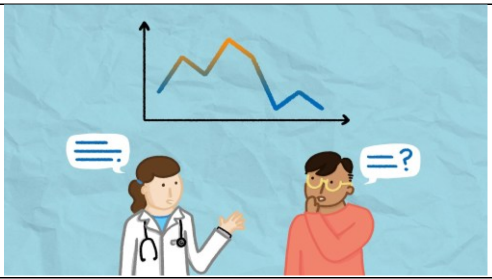	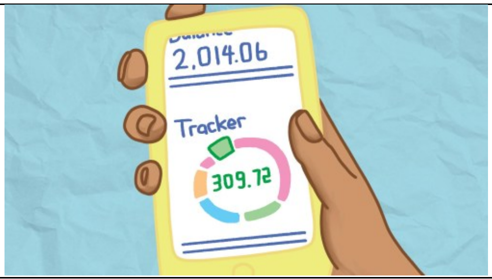
**Action:** A close up of a hand holding a smartphone and scrolling through various types of app features (reminders and streak counters).<br><br>**Voiceover:** Everyone is different when it comes to what kind of app features they prefer. Some people find notifications helpful.<br><br>Other people find ‘gamification’ elements, like streak counters and achievements, especially motivating.	**Action:** A close up of a person talking to their doctor (indicated by speech bubbles) as graph appears overhead.<br><br>**Voiceover:** Others find that sharing data with a healthcare provider or a trusted support person helps keep them on track, as well helping them make sense of changes in how they’re doing over time.	**Action:** A close up of a hand holding a smartphone and scrolling through a budgeting, language, and fitness app.<br><br>**Voiceover:** It can help to think about the apps you use on a daily basis, such as apps for budgeting, learning a language, or keeping track of your diet and fitness - what has helped you use them consistently?

16
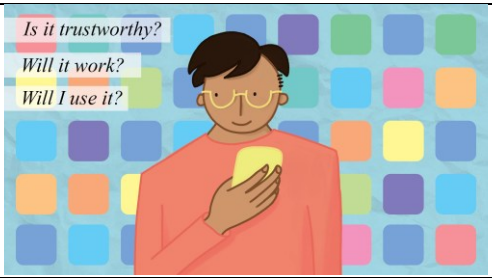
**Action:** A close up of a smiling person holding their mobile phone. The questions in the narration appear on screen behind them.<br><br>**Voiceover:** Many good mental health apps exist, and can help people with bipolar disorder take charge of their own wellbeing.<br><br>Asking yourself,—“is it trustworthy? Will it work? Will I use it?”—will help you navigate your options.<br><br>Your healthcare provider, and certain websites like *MindApps*, *or Psyberguide,* can also assist in this process.<br><br>Finding an app that is trustworthy, effective, and motivating can take time, but your mental health is worth it!
