# Supplementary material for: A Brief Video-Based Intervention to Improve Digital Health Literacy for Individuals With Bipolar Disorder: Intervention Development and Results of a Single-Arm Quantitative Pilot Study
Source: J Particip Med. 2025 May 9;17:e59806. doi: 10.2196/59806 (PMC12102627; doi:10.2196/59806)
Supplement: Multimedia Appendix 2 [file jopm_v17i1e59806_app2.docx]

## Pre-Video

### Demographics

Gender:
◻ Female
◻ Male
◻ Non-binary
◻ Prefer to specify:____________

Age (years): __________________

Marital Status:
◻ Single
◻ In a committed relationship
◻ Common-Law
◻ Married
◻ Divorced
◻ Other:_______

Country of Residence: __________________

How would you describe your ethnic background?

◻ Aboriginal/First Nations/Metis/Inuit,

◻ Black/African/Caribbean,

◻ East Asian,

◻ Latin American,

◻ Middle Eastern,

◻ South Asian,

◻ White/European,
◻ Other/Multiple (please specify): ____________

Highest level of education
◻ Did not finish high school
◻ High school

◻ Some post-secondary (attended college/university but has not completed qualification)
◻ Post-secondary diploma/certificate/associate’s degree
◻ Undergraduate degree
◻ Master’s degree
◻ Ph.D

Main Occupation

◻ Employed Full-Time

◻ Employed Part-Time

◻ Casual Employment

◻ Unemployed

◻ Student Full-Time

◻ Student Part-Time

◻ Volunteer

◻ Retired

◻ Home Duties

◻ Pension (Please specify: ____________)

Type of Bipolar Disorder:

◻ Type I

◻ Type II

◻ Other/don’t know (please elaborate): ___________________________

Are you currently in treatment for your bipolar disorder?

◻ Yes

◻ No

If yes, which of the following treatments are you receiving?

◻ Medication

◻ Counselling/Psychotherapy

◻ Peer support

◻ Other (please specify): ___________________________

### Current use of technology

The term ‘app’ in this survey refers specifically to a mobile app – a software application developed specifically for use on a smartphone device. This section contains questions about your general use of technology and your thoughts about health and wellbeing apps.

Do you use any kind of apps on your smartphone?

- Y/N

Have you ever used an app to help you manage your health and wellbeing?

- Y/N

Have you ever used an app to help you with your bipolar disorder?

- Y/N

If yes,

When choosing a health app, what sources of information do you use to help make your decision? Check all that apply:

◻ Healthcare provider

◻ Other people living with bipolar disorder

◻ Family or friends

◻ App store reviews

◻ Academic journals

◻ Organizations led by people with lived experience of bipolar disorder or other mental health conditions

◻ Government or health organizations

◻ Other (please specify): ___________________________

### Thoughts on mental health apps (mHealth Literacy)

Please rate the degree to which you agree or disagree with these statements (1-5 Likert scale, strongly disagree to strongly agree).

I know how to use smartphone apps to support my health and wellbeing.

I feel motivated to use smartphone apps to support my health and wellbeing.

I am able to find and download a mental health app that fits my needs.

I can tell which apps protect my data and which apps do not.

I know where to find resources which can help me choose mental health apps.

I am able to ask my healthcare provider for support with figuring out what mental health apps might work for me.

### Digital literacy: eHEALS (Norman & Skinner, 2006; adapted text from Hoffman et al., 2020)

How useful do you feel your smartphone is in helping you make decisions about your health?

◻ Not useful at all

◻ Not useful

◻ Unsure

◻ Useful

◻ Very Useful

How important is it for you to be able to access health resources on your smartphone?

◻ Not important at all

◻ Not important

◻ Unsure

◻ Important

◻ Very important

(1-5 Likert scale, strongly disagree to strongly agree)

1. I know how to find helpful health resources on my smartphone
2. I know how to use my smartphone to answer my health questions
3. I know what health resources are available on my smartphone
4. I know where to find helpful health resources on my smartphone
5. I know how to use the health information I find on my smartphone to help me
6. I have the skills I need to evaluate the health resources I find on my smartphone
7. I can tell high quality from low quality health resources on my smartphone
8. I feel confident in using information from my smartphone to make health decisions

## Post-Video

### Video Evaluation

Please rate the degree to which you agree or disagree with these statements (1-5 Likert scale, strongly disagree to strongly agree).

I found the information in this video helpful.

The information provided in the video was relevant to me.

The information in the video is easy to understand.

The video is an appropriate length.

If disagree or strongly disagree: Please specify: “too long” or “too short”

I learned something new from this video.

I would recommend this video to people living with bipolar disorder.

I feel confident that the advice given in the videos is accurate.

Overall, what did you think about the video? (free text)

Are there any additional topics you would have liked to have seen covered in this video? (free text)

### Thoughts on mental health apps (mHealth Literacy)

Please rate the degree to which you agree or disagree with these statements (1-5 Likert scale, strongly disagree to strongly agree).

I know how to use smartphone apps to optimise my health and wellbeing.

I feel motivated to use smartphone apps to optimise my health and wellbeing.

I am able to find and download a mental health app that fits my needs.

I am able to differentiate between apps that protect my data and apps that do not.

I am aware of resources which can help me evaluate mental health apps.

I am able to ask my healthcare provider for support with finding and evaluating mental health apps.

### Digital literacy: eHEALS (Norman & Skinner, 2006; adapted text from Hoffman et al., 2020)

How useful do you feel your smartphone is in helping you make decisions about your health?

◻ Not useful at all

◻ Not useful

◻ Unsure

◻ Useful

◻ Very Useful

How important is it for you to be able to access health resources on your smartphone?

◻ Not important at all

◻ Not important

◻ Unsure

◻ Important

◻ Very important

(1-5 Likert scale, strongly disagree to strongly agree)

1. I know how to find helpful health resources on my smartphone
2. I know how to use my smartphone to answer my health questions
3. I know what health resources are available on my smartphone
4. I know where to find helpful health resources on my smartphone
5. I know how to use the health information I find on my smartphone to help me
6. I have the skills I need to evaluate the health resources I find on my smartphone
7. I can tell high quality from low quality health resources on my smartphone
8. I feel confident in using information from my smartphone to make health decisions
