# Supplementary material for: A Brief Video-Based Intervention to Improve Digital Health Literacy for Individuals With Bipolar Disorder: Intervention Development and Results of a Single-Arm Quantitative Pilot Study
Source: J Particip Med. 2025 May 9;17:e59806. doi: 10.2196/59806 (PMC12102627; doi:10.2196/59806)
Supplement: Multimedia Appendix 3 [file jopm_v17i1e59806_app3.docx]

***Comparison of survey completers and non-completers***

We compared those who dropped out prior to survey completion (*n*=12) and those who completed the survey (*n*=42) using independent t-tests for age and baseline eHEALS score, and Chi-square tests for gender and previous use of BD-related health apps.

The Shapiro Wilks test indicated that the age distributions of survey completers (*W*=0.95, *p*=0.06) and non-completers (*W*=0.95, *p*=0.57) was not significantly different from a normal distribution. The independent t-test indicated that the mean age of survey completers (*M*=38.57, *SD*=11.85) was not significantly different from the mean age of non-completers (*M*=45.50, *SD*=11.60), *t*(52)=1.79, *p*=0.08.

The Shapiro Wilks test indicated that the baseline eHEALS score distribution of survey completers was not significantly different from a normal distribution (*W*=0.96, *p*=0.20), but that the baseline eHEALS score distribution of survey non-completers significantly departed from a normal distribution (*W*=0.85, *p*<0.05). Given the t-test is robust to violation of assumptions, the independent t-test was still run. Findings indicated that the baseline eHEALS score of survey completers (*M*=32.65, *SD*=4.76) was not significantly different from the scores of non-completers (*M*=30.17, *SD*=5.64), *t*(50)=1.52, *p*=0.14.

A maximum likelihood ratio Chi-square test was run to assess whether there was a significant association between gender and successful completion of the survey. This test was chosen since the data set was too small to meet the sample size assumption (>20% of the cells should have expected counts > 5) of the ordinary Chi-square test. The test indicated that there was no significant association between the gender of a survey respondent and their completion of the survey, χ²(3)=2.22, *p*=0.53.

Fisher’s exact test was run to evaluate whether there was a relationship between previous use of BD-related mental health apps and successful completion of the survey among respondents. This test was chosen since the data was in the form of a 2x2 table. The results of the test (*p*=0.51) indicated that there was no significant association between previous use of BD-related mental health apps and successful completion of the survey.

## *eHEALS outliers*

A paired-samples t-test was used to assess if there was a significant difference in the eHEALS scores of survey completers before and after watching the video. After examination of the difference variable box plot, two mild outliers were identified. A sensitivity analysis was completed on the sample excluding outliers (*n*=40). Data showed no evidence of non-normality according to the Shapiro Wilk’s test (*W*=0.96, *p*=0.21) and from visual examination of its histogram and Q-Q plot. The paired-samples t-test indicated that survey completers’ eHEALS scores after watching the video were significantly higher (*M*=33.53, SD=4.61) than their baseline scores (*M*=32.65, *SD*=4.76), *t*(39)=-2.82, *p*<.05, *d*=-0.45.

## *eHEALS missing data*

The analysis was repeated on the full sample, with non-completers’ (*n*=12) post-test eHEALS scores imputed using the last observation carried forward. The difference variable significantly departed from normality according to the Shapiro Wilk’s test (*W*=0.90, *p* <.001) and from visual examination of its histogram and Q-Q plot. Given the t-test is robust to violation of assumptions, the test was still run and indicated that survey completers’ eHEALS scores after watching the video were significantly higher (*M*=32.81, *SD*=5.05) than their baseline scores (*M*=31.91, SD=5.09), *t*(53)=-3.16, *p*<0.05, *d*=-0.43.
